# CLRM-01. MACHINE LEARNING TO UNCOVER SIGNATURES OF VULNERABILITY IN GLIOBLASTOMA UMBRELLA SIGNATURE TRIAL (GUST)

**DOI:** 10.1093/noajnl/vdab112.000

**Published:** 2021-09-21

**Authors:** Sen Peng, Matthew Lee, Nanyun Tang, Manmeet Ahluwalia, Ekokobe Fonkem, Karen Fink, Jeffrey Raizer, Christopher Walker, Harshil Dhruv, Michael Berens

**Affiliations:** 1 The Translational Genomics Research Institute, Phoenix, AZ, USA; 2 Miami Cancer Institute, Miami, Florida, USA; 3 Barrow Neurological Institute, Phoenix, AZ, USA; 4 Baylor Scott & White Health, Texas, USA; 5 Northwestern Medicine, Chicago, Illinois, USA; 6 Karyopharm Therapeutics Inc., Newton, MA, USA

## Abstract

Glioblastoma is characterized by intra- and inter-tumoral heterogeneity. A glioblastoma umbrella signature trial (GUST) posits multiple investigational treatment arms based on corresponding biomarker signatures. A contingency of an efficient umbrella trial is a suite of orthogonal signatures to classify patients into the likely-most-beneficial arm. Assigning optimal thresholds of vulnerability signatures to classify patients as “most-likely responders” for each specific treatment arm is a crucial task. We utilized semi-supervised machine learning, Entropy-Regularized Logistic Regression, to predict vulnerability classification. By applying semi-supervised algorithms to the TCGA GBM cohort, we were able to transform the samples with the highest certainty of predicted response into a self-labeled dataset and thus augment the training data. In this case, we developed a predictive model with a larger sample size and potential better performance. Our GUST design currently includes four treatment arms for GBM patients: Arsenic Trioxide, Methoxyamine, Selinexor and Pevonedistat. Each treatment arm manifests its own signature developed by the customized machine learning pipelines based on selected gene mutation status and whole transcriptome data. In order to increase the robustness and scalability, we also developed a multi-class/label classification ensemble model that’s capable of predicting a probability of “fitness” of each novel therapeutic agent for each patient. Such a multi-class model would also enable us to rank each arm and provide sequential treatment planning. By expansion to four independent treatment arms within a single umbrella trial, a “mock” stratification of TCGA GBM patients labeled 56% of all cases into at least one “high likelihood of response” arm. Predicted vulnerability using genomic data from preclinical PDX models correctly placed 4 out of 6 models into the “responder” group. Our utilization of multiple vulnerability signatures in a GUST trial demonstrates how a precision medicine model can support an efficient clinical trial for heterogeneous diseases such as GBM.

